# Structural phase transitions in Bi2Se3 under high pressure

**DOI:** 10.1038/srep15939

**Published:** 2015-11-02

**Authors:** Zhenhai Yu, Lin Wang, Qingyang Hu, Jinggeng Zhao, Shuai Yan, Ke Yang, Stanislav Sinogeikin, Genda Gu, Ho-kwang Mao

**Affiliations:** 1 Center for High Pressure Science and Technology Advanced Research, Shanghai, 201203, People’s Republic of China; 2 State Key Laboratory of Superhard Materials, Jilin University, Changchun 130012, People’s Republic of China; 3 Natural Science Research Center, Academy of Fundamental and Interdisciplinary Sciences, Harbin Institute of Technology, Harbin 150080, People’s Republic of China; 4 Shanghai Institute of Applied Physics, Chinese Academy of Sciences, Shanghai 201203, People’s Republic of China; 5 High Pressure Collaborative Access Team, Geophysical Laboratory, Carnegie Institution of Washington, Argonne, Illinois 60439, United States of America; 6 Condensed Matter Physics and Materials Science Department, Brookhaven National Laboratory, Upton, New York 11973, United States of America; 7 Geophysical Laboratory, Carnegie Institution of Washington, Washington, DC 20015, United States of America

## Abstract

Raman spectroscopy and angle dispersive X-ray diffraction (XRD) experiments of bismuth selenide (Bi_2_Se_3_) have been carried out to pressures of 35.6 and 81.2 GPa, respectively, to explore its pressure-induced phase transformation. The experiments indicate that a progressive structural evolution occurs from an ambient rhombohedra phase (Space group (SG): *R*-3*m*) to monoclinic phase (SG: *C*2/*m*) and eventually to a high pressure body-centered tetragonal phase (SG: *I*4/*mmm*). Evidenced by our XRD data up to 81.2 GPa, the Bi_2_Se_3_ crystallizes into body-centered tetragonal structures rather than the recently reported disordered body-centered cubic (BCC) phase. Furthermore, first principles theoretical calculations favor the viewpoint that the *I*4/*mmm* phase Bi_2_Se_3_ can be stabilized under high pressure (>30 GPa). Remarkably, the Raman spectra of Bi_2_Se_3_ from this work (two independent runs) are still Raman active up to ~35 GPa. It is worthy to note that the disordered BCC phase at 27.8 GPa is not observed here. The remarkable difference in atomic radii of Bi and Se in Bi_2_Se_3_ may explain why Bi_2_Se_3_ shows different structural behavior than isocompounds Bi_2_Te_3_ and Sb_2_Te_3_.

Topological insulators (TIs) are electronic materials that have a bulk band gap like ordinary insulators, but feature conducting states on their surface. Besides the importance of theoretical investigation in condensed matter physics, TIs also have various actual applications in the fields of spintronics^1^,^2^, quantum computation^3^,^4^ and thermoelectric energy conversion^5^,^6^. As excellent thermoelectric materials, Bi_2_Se_3_, Bi_2_Te_3_, and Sb_2_Te_3_ were extensively studied in the 1950s and 1960s. Nevertheless, some basic physical properties still remain unexplored. For example, the A_2_B_3_ (A = Sb, Bi; B = Se, Te) series such as Bi_2_Te_3_, Sb_2_Te_3_, and Bi_2_Se_3_ have exotic gapless surface states^7^,^8^. These were shown to belong to a class of interesting three-dimensional TIs^9^,^10^. The Bi_2_Se_3_ is an ideal candidate for studying room temperature topological insulating behavior, which has a topologically nontrivial band gap of 0.3 eV. This is much larger than the room temperature energy scale^11^.

It is of fundamental significance to explore the crystal structural evolutions of TIs under some controlled external conditions. External pressure is well known as a powerful method to tune atomic arrangements and the consequential properties of materials. *In situ* high-pressure X-Ray diffraction (XRD) investigations on two kinds of TIs (Bi_2_Te_3_ and Sb_2_Te_3_) showed that both exhibit the same crystal structural transformation sequence of *R*-3*m* (CN = 6) → *C*2/*m* (CN = 7) → *C*2/*c* (CN = 8) → *Im*-3*m* (CN = 8)^12^,^13^ with a step-increasing coordination number of Bi atoms. The Bi_2_Se_3_ is isostructural to Bi_2_Te_3_ and Sb_2_Te_3_ and thus it is natural to speculate that Bi_2_Se_3_ would follow the same sequence of transformation under high pressure.

The structural variations of Bi_2_Se_3_ have been studied previously. Angular dispersive powder XRD and Raman spectrum measurements on Bi_2_Se_3_ were carried out using a Merrill-Bassett diamond anvil cell (DAC) by Vilaplana *et al.*^14^. whose results suggest a pressure-induced structural phase transition sequence of *R*-3*m* (CN = 6) → *C*2/*m* (CN = 7) → *C*2/*c* (CN = 8) → *Im*-3*m* (CN = 8) structures at 10, 20, and 28 GPa, respectively. The Raman modes of Bi_2_Se_3_ were inactive over 27.8 GPa suggesting that Bi_2_Se_3_ crystallized into a disordered body-centered cubic (BCC) (*Im*-3*m*) structure above that pressure. Concurrently, high pressure XRD studies on Bi_2_Se_3_ have also been performed by other research groups^15^−^17^. However, the assignments of the high-pressure phase for Bi_2_Se_3_ are controversial from different literatures as shown in Fig. 1(a). To the best of our knowledge, at least seven crystallographic models for Bi_2_Se_3_ were reported under different external conditions (Fig. 1(b)). In addition to the remarkable effect of high pressure on the crystal structures of Bi_2_Se_3_, previous studies have also shown that a pressure-induced superconductivity in Bi_2_Se_3_ occurs at ~11 GPa^17^.

Given the above mentioned confusing experimental and calculation results on the high-pressure polymorphism of Bi_2_Se_3_, our work is motivated to deal with two major issues: (1) The first clarifies the ambiguity that which phase (*C*2/*m* or the resemblance *C*2/*c* phase) lies in Bi_2_Se_3_ around 10 GPa because *C*2/*c* is a subgroup of space group *C*2/*m*. (2) The second clarifies the high-pressure phase of Bi_2_Se_3_, which depends on whether we could identify the weak diffraction peaks located in the lower angles. As mentioned above, the crystallographic polymorphisms under high pressure are still unclear due to a lack of systematic work. In addition to the above issues, we have carried out high pressure synchrotron XRD studies on Bi_2_Se_3_ to investigate the pressure-induced structural transformation in Bi_2_Se_3_. Furthermore, the high pressure Raman spectra measurement on Bi_2_Se_3_ was also performed with different pressure-transmitting mediums (PTM) to promote the clarification of polymorphisms in Bi_2_Se_3_. Finally, we used theoretical calculations to confirm the experimental data regarding structural stability of high-pressure phase of Bi_2_Se_3_

## Results

The phase quality of our samples was first confirmed with high-resolution synchrotron XRD (Fig. S1). The selected angular dispersive XRD patterns collected from ambient pressure to 81.2 GPa are shown in Fig. 2 (a) with some additional peaks of the pressure marker Au. It can be seen that two phase transitions occur at near ~11 and ~30 GPa from the obviously changing of the diffraction peaks. The more visual two-dimensional (2D) XRD image of the new phase at 81.2 GPa for Bi_2_Se_3_ is shown in Fig. 2 (b). The Rietveld refined diffraction patterns of the three phases are depicted in Fig. 3. The peak fitting processes will be analyzed in more detail below. The XRD pattern of the low-pressure phase of Bi_2_Se_3_ was readily fitted based on the rhombohedral phase model (space group *R*-3*m*) below 11.4 GPa (Fig. 3(a)). The Bi_2_Te_3_, Sb_2_Te_3_, and Bi_2_Se_3_ all crystallize into *R*-3*m* crystal structures at ambient conditions. An electronic topological transition (ETT) in the *R*-3*m* phase of Bi_2_Te_3_ (α-Bi_2_Te_3_) was observed, which leads to a pronounced change in the *c*/*a* ratio at ~3 GPa^18^. The ETT or Lifshitz transition, which interprets the transitions between different Fermi surface topologies. The ETT could be induced not only by compression or alloying, but also by temperature and magnetic fields, which are able to tune the electronic structure of the material. Correspondingly, The ETT causes anomalous behavior in the thermodynamic, transport and elastic properties^19^. Similar variations in the *c*/*a* ratio were also observed in Bi_2_Se_3_ from our present experimental XRD results. The pressure dependence of the axial ratio (*c*/*a*) for the *R*-3*m* phase of Bi_2_Se_3_ is reported in Fig. S3. Our current measurement indicates that the pressure-induced ETT in Bi_2_Se_3_ occurred at ~3 GPa, the profile of which is different from that in ref. 14. (see supplementary information).

**Figure 1:**
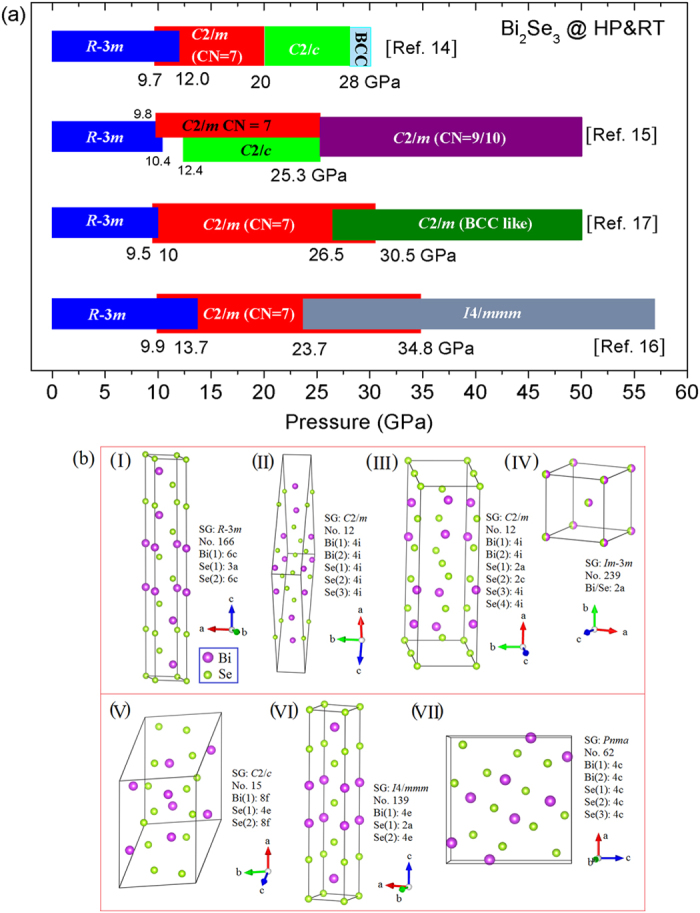
(**a**) Phase diagram of Bi_2_Se_3_ under room temperature with increasing pressure referred from several adapted literatures [14, 15, 16, 17]. (**b**) Selected crystallographic structure models of Bi_2_Se_3_ under different external conditions.

**Figure 2:**
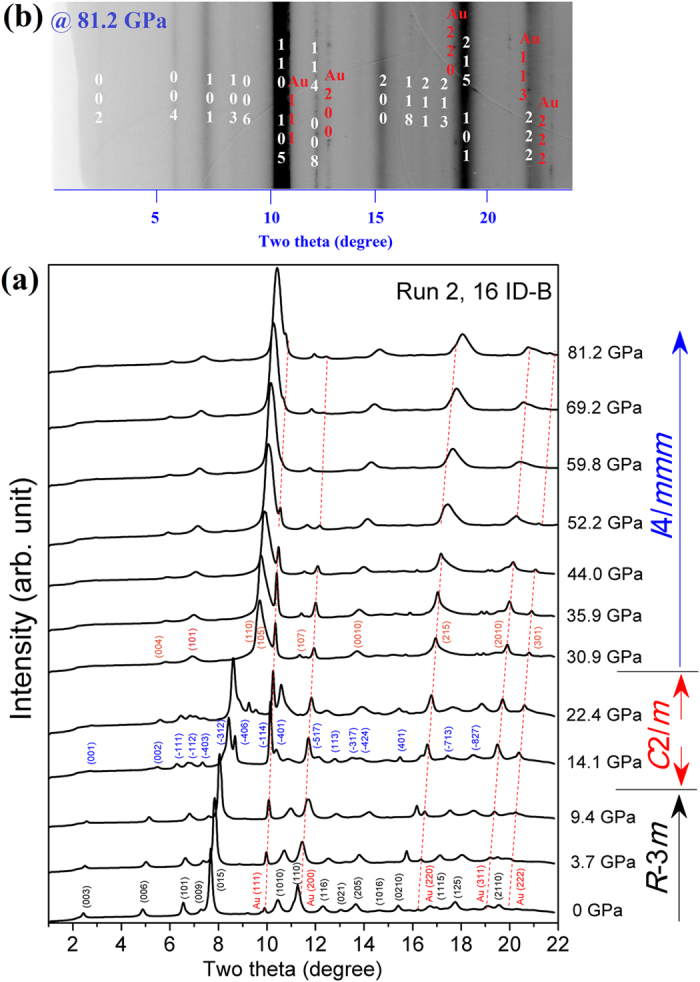
(**a**) The selected angle dispersive XRD patterns of Bi_2_Se_3_ under various pressures at room temperature from ambient pressure up to 81.2 GPa. The diffraction peaks of Au were marked with dashed lines. (**b**)The cake two-dimensional (2D) image of Bi_2_Se_3_ under 81.2 GPa.

**Figure 3:**
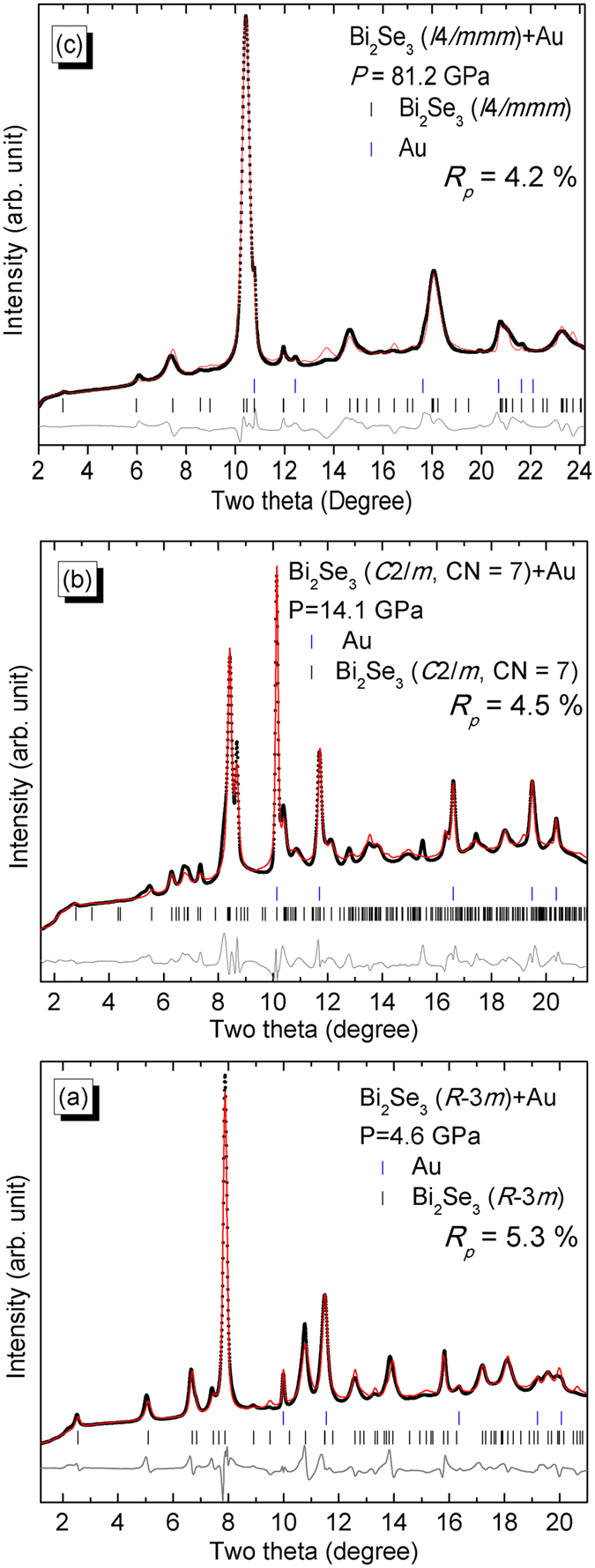
Typical Rietveld refinement results of Bi_2_Se_3_ under (**a**) 4.6, (**b**) 14.1, and (**c**) 81.2 GPa. The experimental and simulated data were symbolled with black solid sphere and red line. The solid short vertical lines show the positions of the allowed Bragg reflections for Bi_2_Se_3_ and Au. The difference between the observed and the fitted XRD patterns were shown with a line at the bottom of the diffraction peaks.

A pressure-induced structural phase transition in Bi_2_Se_3_ occurs at ~10 GPa and this has been intensively investigated over the past years. Nevertheless, an ambiguity in the space group assignment (*C*2/*m* or *C*2/*c*) of this new high-pressure phase still exists. The main difference in these two space group candidates is that the *C*2/*m* possesses mirror-plane symmetry and the *C*2/*c* possesses slide-plane symmetry. To emphasize, the space group of the *C*2/*m* phase Bi_2_Se_3_ still has two types due to different assignments for the Bi and Se atoms on the Wyckoff positions. The corresponding crystallographic models can be found in Fig. 1(b). The XRD pattern of Bi_2_Se_3_ only has one merged diffraction peak located at the *d*-spacing position between 2.7939 Å (2*θ* = 8.346°) and 2.6019 Å (2*θ* = 8.963°) under a quasihydrostatic pressure of ~14 GPa^16^, while our experimental result shows that this merged diffraction peak splits into two separate diffraction peaks under nonhydrostatic conditions (Fig. S4). After comparing our experimental XRD pattern of Bi_2_Se_3_ at around 14 GPa and the calculated XRD patterns with *C*2/*c* (CN = 8) and *C*2/*m* (CN = 7) space groups (Fig. S4), the *C*2/*m* (CN = 7) was selected as the more favorable structure for Bi_2_Se_3_ at this pressure. A typical Rietveld refinement of our experimental XRD pattern for the *C*2/*m* phase Bi_2_Se_3_ is shown in Fig. 3(b).

The high-quality angle-dispersive synchrotron XRD patterns of Bi_2_Se_3_ in this study enabled us to differentiate weak diffraction peaks located at lower angles. Therefore, we concluded that Bi_2_Se_3_ actually crystallizes into a body-centered tetragonal structure when it is pressurized to 30.9 GPa and even up to 81.2 GPa. The angular dispersive XRD data can be unambiguously fitted into the space group *I*4/*mmm* for the best results. The Rietveld refinement of this body-centered tetragonal structure is shown in Fig. 3(c). Another series of duplicate high pressure XRD measurement was performed at the BL15U1 beamline of the Shanghai Synchrotron Radiation Facility (SSRF). The results are shown in Fig. S2 and confirm our conclusions.

**Figure 4:**
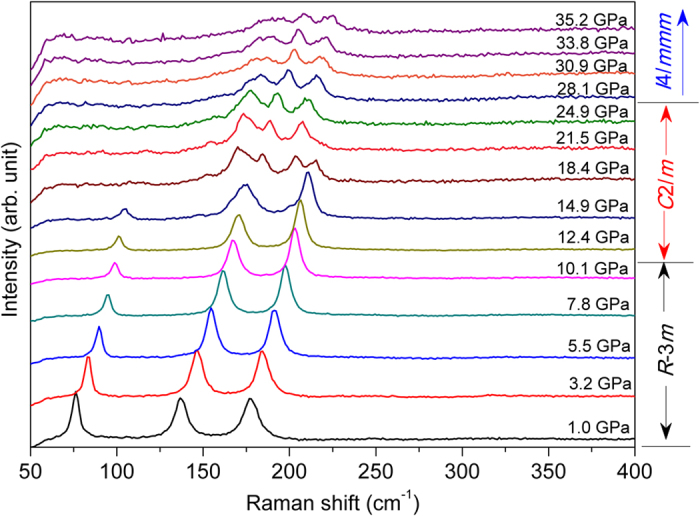
Pressure dependence of Raman spectra of Bi_2_Se_3_ during compression using 4:1 methanol-ethanol mixture.

To verify our speculation on the crystallographic structural phase transition sequence of Bi_2_Se_3_ under high pressure, Raman scattering spectroscopy, which is a more sensitive technique in investigating vibrational, rotational, and other low-frequency modes of materials, was employed to characterize the pressure-induced structural phase transition. Under ambient conditions, the Bi_2_Se_3_ crystallizes into a rhombohedral structure with space group *R*-3*m*. It contains five atoms in a primitive unit cell. Therefore, there are four Raman-active modes (2A_1g_ + 2E_g_) and four IR-active modes (2A_2u_ + 2E_u_). A 4:1 methanol-ethanol mixture was used as the PTM in the Raman experiment, and several selected Raman spectra from the Stokes contribution in Run 1 are shown in Fig. 4. The Raman spectrum from the *R*-3*m* phase of Bi_2_Se_3_ was located at the lower panel of Fig. 4. The present measured Raman spectrum of the *R*-3*m* phase for Bi_2_Se_3_ agrees nicely with the experimental results in ref. 14.

According to the group theory analysis and the literature reported results^20^−^22^, the Raman peaks located at ~72.4, ~133.2 and 174.3 cm^−1^ are identified as the 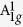, 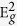 and 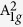 vibrational modes, respectively. As the pressure increasing, we found from Fig. 4 that all three Raman peaks shift to higher wavenumbers (blue shift). The Raman peaks representing the 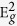 and 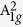 modes appear as a single sharp peaks up to about 10 GPa. Those peaks are quite broad beyond 10 GPa and new Raman peaks appear, indicating a structural change has occurred. In line with the XRD experiments, this is attributed to the structural phase transformation from the hexagonal (*R*-3*m*) to the monoclinic phase (*C*2/*m*). The onset of the structural phase transition is assigned around 10.1 GPa on the basis of Raman observation. Upon further compression, we observed that the Raman spectrum above ~28 GPa is unambiguously different than that of the *C*2/*m* phase. Though the Raman peaks become more and more broaden as the pressure increasing. The Raman spectra of the new phase of Bi_2_Se_3_ are still discernable up to ~35 GPa. The above phenomena faultlessly support our XRD result that the *C*2/*m* phase of Bi_2_Se_3_ transforms to the tetragonal phase (*I*4/*mmm*) above 30.9 GPa. The authors in ref. [14]. commented that Bi_2_Se_3_ follows the same structural transformation sequence as observed in Bi_2_Te_3_ and Sb_2_Te_3_ isostructures and finally transforms into a disordered BCC structure as deduced by their result that no Raman active modes can be detected at 27.8 GPa. Their conclusion is inconsistent with our data.

Corresponding to our current nonhydrostatic XRD measurements, the Raman scattering experiment for Bi_2_Se_3_ here was also performed without PTM. The Raman spectra of Bi_2_Se_3_ under various pressures without PTM are shown in Fig. S6. Comparing to the results under hydrostatic conditions in Fig. 4, we note that the profile of Raman spectra is nearly identical to what we collected with a pressure medium of 4:1 methanol-ethanol mixture. The Raman result of Bi_2_Se_3_ in this study again confirms our high pressure XRD analysis.

**Figure 5:**
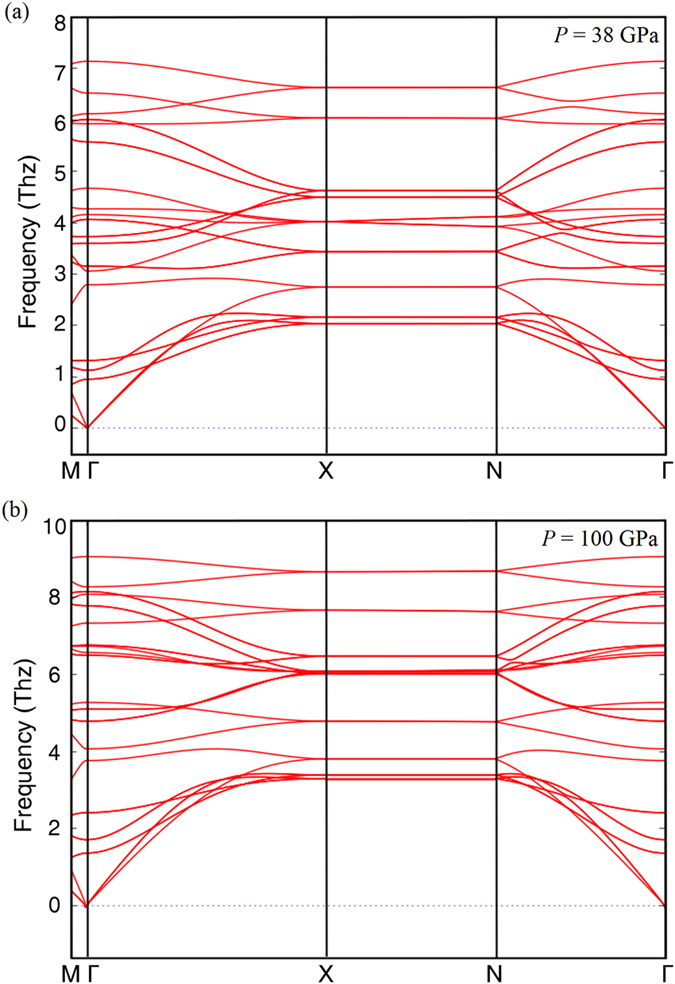
Selected pressure dependence of phonon dispersion for *I*4*/mmm* phase of Bi_2_Se_3_.

To confirm the mechanical stability of the high-pressure phase (space group: *I*4/*mmm*) of Bi_2_Se_3_, we carried out the phonon calculations under the framework of density functional theoretical (DFT). The results are listed in an evolution of increasing pressure (Fig. 5), where the compression of the system results in a blue shift of the all phonon mode frequencies, reproducing the experiments described above. Even up to 100 GPa, no negative modes appear in the phonon dispersion curves. This indicates that this structure is mechanically stable. The stability illustrated via phonon analysis is consistent with our XRD and Raman data.

## Discussion

The high-pressure phase of Bi_2_Se_3_ transformed from the *C*2/*m* phase has three debating candidates, which are *Im*-3*m*^14^, *C*2/*m* (CN = 9/10)^15^ and *I*4/*mmm*^16^. To clarify the polymorphism in Bi_2_Se_3_, we further enumerate the experimental XRD data around 30 GPa from this work and calculated XRD results with the three candidate space groups reported in the literatures (Fig. S5). The simulated XRD patterns with space groups *C*2/*m* (CN = 9/10) and *Im*-3*m* could not match the experimental XRD data collected here.

The A_2_B_3_ (A = Sb, Bi; B = Se, Te) series compounds such as Bi_2_Te_3_, Sb_2_Te_3_, and Bi_2_Se_3_ were reported to exhibit topological properties^7^,^11^. Therefore it is natural to check if the isocompound Sb_2_Se_3_ exhibits the same properties. Recently, Sb_2_Se_3_ was reported to transform from a non-topological state into a topological insulation state above ~2 GPa and forming a disordered BCC structure above 51 GPa^23^. At ambient conditions, the radii of the Se, Te, Sb and Bi atoms are 1.15, 1.40, 1.45, and 1.60 Å, respectively^24^, from which we could found that Bi and Se atoms possess the biggest atomic radii difference among the above mentioned four atoms. Therefore, a certain pressure could not make these Bi and Se atoms resemble, this might be why the pressure-induced disordered substitution structure (*Im*-3*m*) observed in Bi_2_Te_3_, Sb_2_Te_3_ and Sb_2_Se_3_ did not appear in Bi_2_Se_3_ until up to 81.2 GPa from this work.

**Figure 6:**
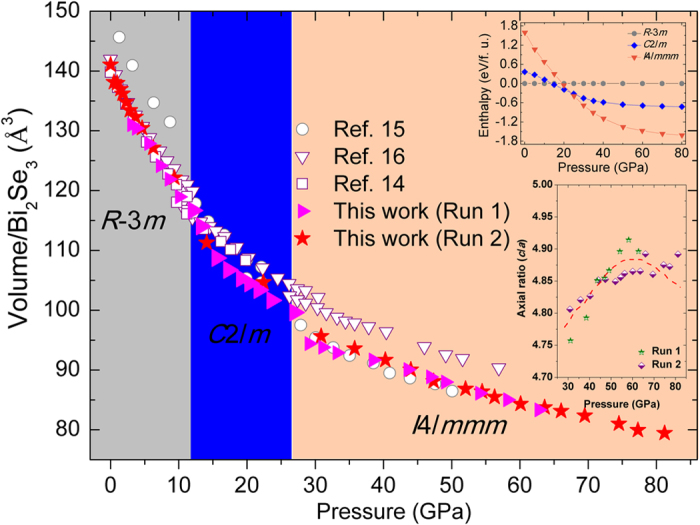
Pressure dependence of lattice volume for Bi_2_Se_3_. Open symbol data were taken from ref. 14,15 and 16. Data from this work were marked with solid legend. Pressure dependence of *c*/*a* ratio for *I*4*/mmm* phase was shown in bottom-right of inset, where dashed lines were guide for eyes. Enthalpy curves (relative to *R*-3*m* phase) for high pressure phases as a function of pressure (top-right of inset).

The equation of state for Bi_2_Se_3_ is shown in Fig. 6. The unit cell volume for *R*-3*m* phase obtained from this work matches the literature results in refs 14 and 16 well, but exhibits smaller volume than that in ref. 15 at the same pressure. This contradiction may be due to the different preparation techniques used to synthesize the samples. Given the XRD data from this work and previous reported results, the pressure points of the phase transitions in Bi_2_Se_3_ are assigned to ~10 (*R*-3*m* → *C*2/*m*) and ~25 (*C*2/*m* → *I*4/*mmm*) GPa, respectively. Although the high pressure polymorphism of Bi_2_Se_3_ still remains under debate, it is generally accepted that the pressure-induced structural phase transition in Bi_2_Se_3_ possess the feature of a first order phase transition.

The present enthalpy calculations (top-right inset in Fig. 6) show the structural stability of different phases under high pressure, which are in good agreement with the experimental results. The pressure dependence of the *c*/*a* ratio for the *I*4/*mmm* phase Bi_2_Se_3_ is plotted in the bottom-right inset of Fig. 6. A unit cell of the *I*4/*mmm* phase Bi_2_Se_3_ contains ten atoms as shown in Fig. 1(b). It can be seen from the bottom-right inset of Fig. 6 that the value of the *c*/*a* ratio shows dome-shaped pressure dependence. It first increases and reaches a maximum of 4.90 near 60 GPa and then decreases as the pressure increasing. The present pressure dependence of the *c*/*a* ratio for *I*4/*mmm* phase for Bi_2_Se_3_ is consistent with the above XRD and Raman data. To emphasize, the *c*/*a* ratio should approach 5.00 if the *I*4/*mmm* phase transformed into the BCC phase.

In conclusion, the joint first-principles theoretical calculation and experimental measurements were performed to investigate the pressure-induced structural phase transitions in Bi_2_Se_3_. High pressure XRD results reveal two pressure-induced structural phase transitions in Bi_2_Se_3_, which did not follow the same transformation sequence as that in the isostructural compounds Bi_2_Te_3_ and Sb_2_Te_3_. The Raman spectra results rule out the previous hypothesis that Bi_2_Se_3_ transforms into a disordered BCC structure at ~30 GPa. The present experimental XRD measurement and DFT calculations confirm the structural stability of the high-pressure *I*4/*mmm* phase.

## Materials & Methods

### Crystal growth and preparation

The Bi_2_Se_3_ single crystals in this work were grown by a unidirectional solidification method with slowly cooling down. The samples were ground in a mortar to obtain a fine powder sample used in the following high resolution synchrotron XRD, high pressure angle dispersive XRD and Raman experiments.

### High resolution synchrotron XRD

First, the phase purity of sample (Bi_2_Se_3_) used in this work was confirmed by using high resolution synchrotron XRD measurement. High resolution synchrotron XRD data were collected using the powder diffractometer at 11 BM-B beamline of Advanced Photon Source at Argonne National Laboratory. The wavelength was fixed at 0.4124 Å. The wavelength was calibrated using Si 640c as the standard. The samples were finely ground and housed in glass capillaries that were continuously rotated during the measurements.

### High pressure synchrotron angular dispersive XRD

The high pressure synchrotron XRD patterns were obtained using a symmetric diamond anvil cell with 200 μm culet diameter. A rectangle foil of rhenium was pre-indented down to a thickness of 35 μm by compressing the two diamond anvils. A 60-μm gasket hole was made by drilling at the center of indention in the foil. The powdered sample was put in the gasket hole without pressure medium. After the sample was pressed by the anvil, some gold powder was loaded on the flat of the sample to calculate the value of the pressure in the sample chamber^25^. The angular dispersive powder XRD patterns were taken with a Mar3450 detector using synchrotron radiation beams monochromatized to a wavelength of 0.4066 Å at 16 ID-B beamline of the Advanced Photon Source at Argonne National Laboratory. An independent high pressure XRD experiment was also performed at BL15U1 beamline at Shanghai Synchrotron Radiation Facility (SSRF) using a monochromatic beam of 0.6199 Å. The two-dimensional image plate patterns were integrated to the one-dimension patterns by using the Fit2D software^26^. The resulting diffraction patterns were fitted via Rietveld refinement through a GSAS package^27^.

### Raman spectroscopy under high pressure conditions

The Raman spectroscopy investigation on Bi_2_Se_3_ under high pressure was carried out using a commercial Renishaw Raman spectroscopy system in the backscattering configuration excited with a He/Ne laser (λ = 632.8 nm). The spectra resolution is as small as 1 cm^−1^, and the lowest available frequency is 50 cm^−1^. Two independent high-pressure Raman experiments were implemented on Bi_2_Se_3_. The methanol-ethanol mixture was used as PTM in Run 1, and no PTM was used in Run 2.

### Density functional calculation

In this study, first-principles calculations^28^−^30^ were performed in the framework of density functional theory with the Vienna *ab initio* simulation package^31^. The generalized gradient approximation under Perdew-Wang parameterization^32^ was implemented to describe the exchange correlation functions. The Projected-augmented wave potentials^33^,^34^ were used with 24 valence electrons for Bi (6s^2^6p^3^) and 8 for Se (4s^2^4p^4^). A plane-wave basis set with kinetic energy cut-off of 300 eV was found sufficient to converge a total energy less than 10^−6^ eV and force acting on each atom less than 0.01 eV/Å. One Bi_2_Se_3_ unit cell (10 atoms, space group *I*4/*mmm*) was used for calculating electronic structures. The Brillouin zone is sampled by a Monkhorst mesh^35^ of 5 × 5 × 1 k-points, providing totally 9 irreducible k-points. A more dense mesh of k-points (10 × 10 × 2) mesh was tested, and the energy difference with the 5 × 5 × 1 mesh is less than the energy convergence criterion. Therefore the smaller mesh was used for all of our calculations. Hydrostatic pressure is applied by adding Pulay stress to the diagonal elements of the stress tensor. At each pressure, the unit cell is fully optimized for atomic position, cell shape and cell volume.

We carried out supercell approach (2 × 2 × 1 supercell) within the finite displacement method for the phonon calculations^36^. Force constants are calculated using the Moore–Penrose pseudo-inverse by fitting symmetry reduced elements of force constants to the linear relations between atomic forces and atomic displacements by the Phonopy package^37^.

## Additional Information

**How to cite this article**: Yu, Z. *et al.* Structural phase transitions in Bi_2_Se_3_ under high pressure. *Sci. Rep.* **5**, 15939; doi: 10.1038/srep15939 (2015).

## Supplementary Material

Supplementary Information
