# Supplementary material for: Structural phase transitions in Bi2Se3 under high pressure
Source: Sci Rep. 2015 Nov 2;5:15939. doi: 10.1038/srep15939 (PMC4629115; doi:10.1038/srep15939)
Supplement: Supplementary Information [file srep15939-s1.pdf]

Structural phase transitions in Bi_2Se_3 under high pressure

Zhenhai Yu[1], Lin Wang[1,2,7]* Qingyang Hu[1,7], Jinggeng Zhao[3], Shuai Yan[4], Ke Yang[4], Stanislav Sinogeikin[5], Genda Gu[6] & Ho-kwang Mao[1,5,7]

[1]Center for High Pressure Science and Technology Advanced Research, Shanghai, 201203, People's Republic of China,

[2]State Key Laboratory of Superhard Materials, Jilin University, Changchun 130012, People's Republic of China,

[3]Natural Science Research Center, Academy of Fundamental and Interdisciplinary Sciences, Harbin Institute of Technology, Harbin 150080, People's Republic of China,

[4]Shanghai Institute of Applied Physics, Chinese Academy of Sciences, Shanghai 201203, People's Republic of China,

[5]High Pressure Collaborative Access Team, Geophysical Laboratory, Carnegie Institution of Washington, Argonne, Illinois 60439, United States of America,

[6]Condensed Matter Physics and Materials Science Department, Brookhaven National Laboratory, Upton, New York 11973, United States of America,

[7]Geophysical Laboratory, Carnegie Institution of Washington, Washington, DC 20015, United States of America,

* Corresponding author, Tel: 1(630)252-6763 (office), Fax 1(630)252-9303.

E-mail address: wanglin@hpstar.ac.cn, or lwang@carnegiescience.edu (Lin Wang).

(1) High resolution XRD pattern of Bi_2Se_3 under ambient conditions

High resolution synchrotron X-ray powder diffraction data were collected using the powder diffractometer at 11 BMB beamline of Advanced Photon Source at Argonne National Laboratory. The wavelength was set at 0.4124 Å. The wavelength was calibrated using Si 640c as a standard. The samples were finely ground and housed in glass capillaries that were continuously rotated during the measurements. Fig. S1 depicts a typical Rietveld refinement of *R*-3*m* phase of Bi_2Se_3 under ambient conditions. The XRD pattern of Bi_2Se_3 at ambient conditions was indexed with a rhombohedral structure (*R*-3*m*, No. 166) and with hexagonal representation lattice parameters a = 4.1408(9) Å, c = 28.647(6) Å, and V_0 = 425.4(2) Å^3. The lattice parameters refined from this work are good agreement with those from Nakajima et al. (a = 4.143 Å, c = 28.636 Å, and V_0 = 425.673 Å^3).

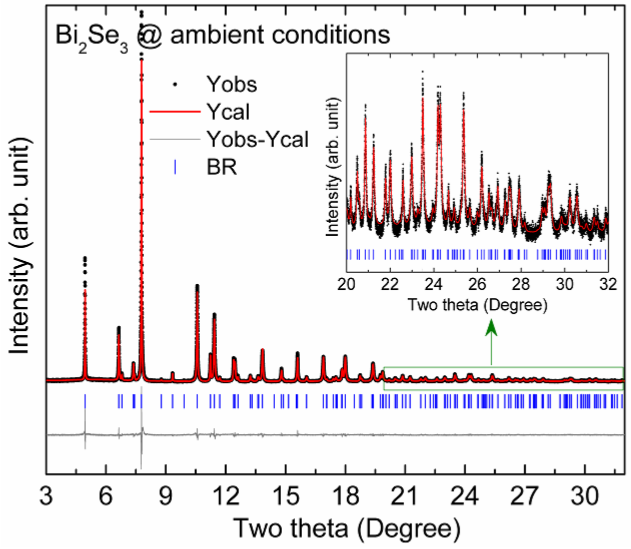

Figure S1. High resolution synchrotron X-ray diffraction profiles for Bi_2Se_3 under ambient conditions. The solid spheress are the observed data and the solid lines are the calculated diffraction patterns. The positions of the space group allowed reflections are indicated with vertical solid short lines. The inset shows the quality of the fit and data at higher angles.

(2) Angle dispersive XRD patterns of Bi_2Se_3 collected at BL15U1 beamline of Shanghai Synchrotron Radiation Facility

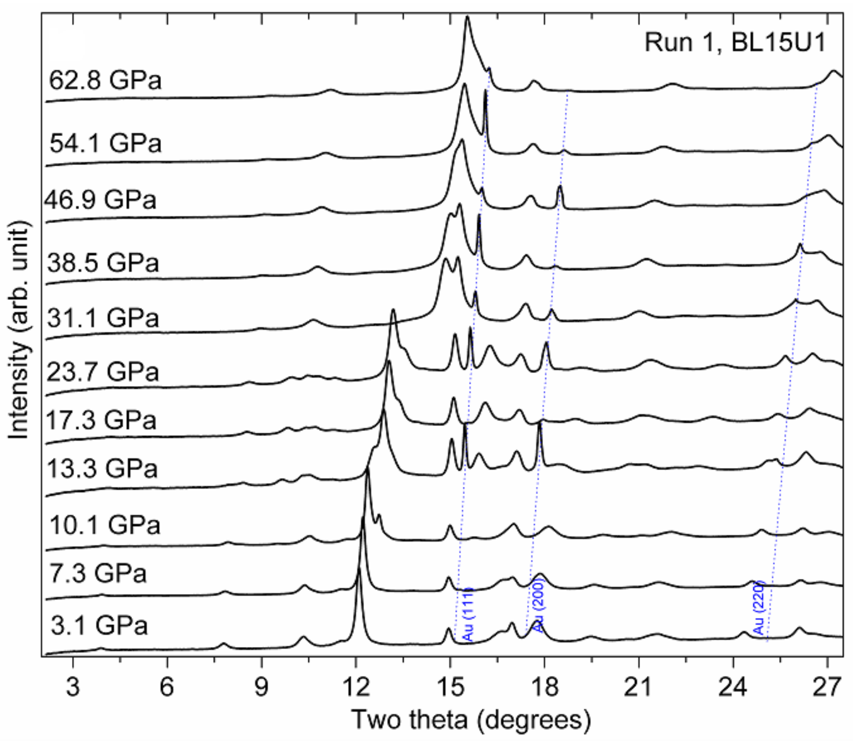

Fig. S2. Angle dispersive XRD patterns of Bi_2Se_3 under various pressures at room temperature collected at BL15U1 beamline of Shanghai Synchrotron Radiation Facility.

(3) Pressure induced electronic topological transition (ETT) in *R*-3*m* phase of Bi_2Se_3

The lattice parameters *a* and *c* as a function of pressure for *R*-3*m* phase of Bi_2Se_3 were obtained from the Rietveld refinement. The pressure dependence of the axial ratio (*c*/*a*) for *R*-3*m* phase of Bi_2Se_3 is reported in Fig. S2. The axial ratio decrease as the pressure increase. However the slope of the axial ratio versus pressure is different. Our present measured results indicate that pressure induced ETT in Bi_2Se_3 occurred around 3 GPa. The experimentally observed pressure-induced ETT in Bi_2Se_3 was also confirmed by Vilaplana *et al*.. They observed the pressure-induced ETT in Bi_2Se_3 around 5 GPa. However the axial ratio of *c/a* in Vilaplana *et al*.'s results experiences minor changes among 3.4 to 8.4 GPa as shown in Fig. 3 with open symbols. A 4:1 methnol-ethnol mixture was used as PTM in Vilaplana et al.'s experiment [14]. No PTM was used in this work. We attribute the discrepancies to the effects of the different PTM used in the high-pressure experiments.

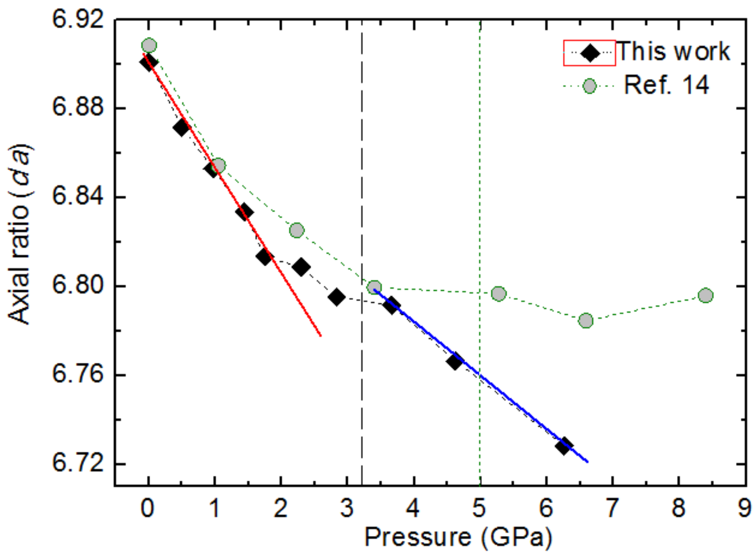

Fig. S3. The pressure dependence of axial ratio (*c/a*) for *R*-3*m* phase of Bi_2Se_3

(4) Assignment of Wyckoff position in *C*2/*m* phase of A_2B_3-type topological insulator

Respecting the high pressure phase with space group of *C*2/*m* for A_2B_3-type topological insulator, there are three cases of assignment of Wyckoff position from the literature, (I) two types of A atoms and three types B atoms located at five nonequivalent crystallographic 4i Wyckoff position; (II) Two types of nonequivalent A atoms was situated at 4i position, the wyckoff positions for the four nonequivalent B atoms are 2a, 4i, 2c, and 4i, respectively; (III) A and B atoms could reach a state with disordered solution due to the close atomic radii of A and B atoms such as Bi, Sb and Te. The Wyckoff positions for A/B atoms are 2a and 4i, respectively.

(5) Comparison of experimental and simulated XRD patterns of Bi_2Se_3 ~ 14 GPa

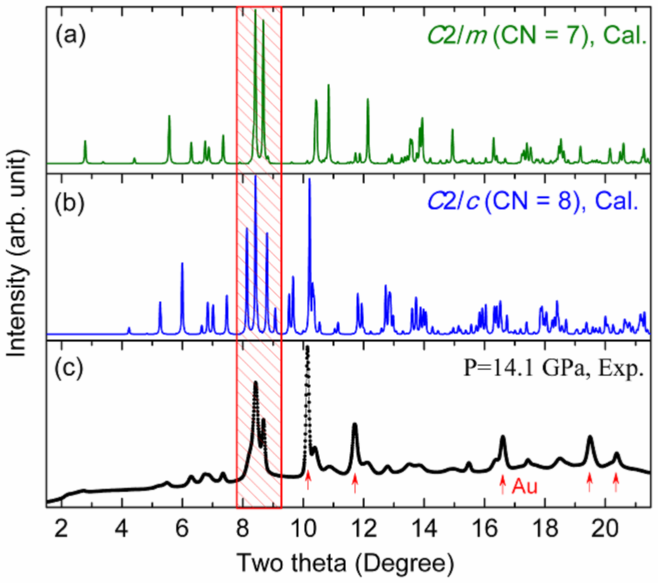

Fig. S4 Angles dispersive XRD pattern of Bi_2Se_3 ~ 14 GPa from this work (the diffraction peaks of Au were marked with red arrows) and the simulated XRD patterns with space group *C*2/*c* and *C*2/*m*.

(6) Comparison of experimental and simulated XRD patterns of Bi_2Se_3 ~ 30 GPa

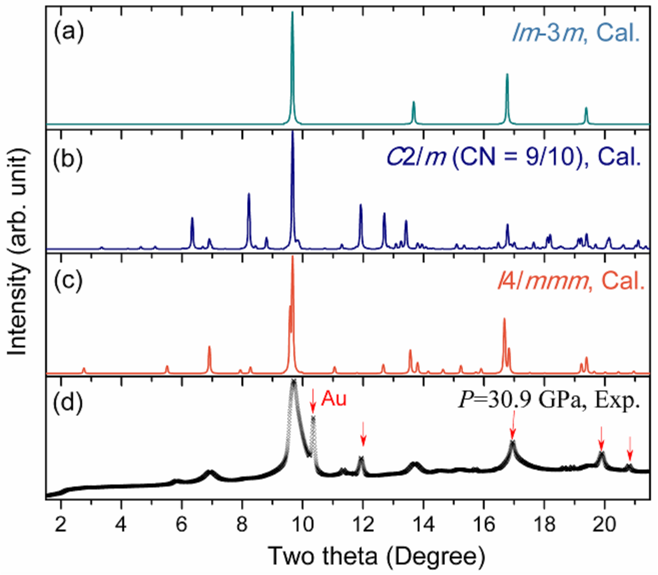

Fig. S5. The experimental and simulated XRD patterns of Bi_2Se_3 with different polymorphism under high pressure around 30 GPa. The lattice parameters of *C*2/*m*, *I*4/*mmm* and *Im*-3*m* phase of Bi_2Se_3 were referenced from Ref. (14-17)

(7) Pressure dependence of Raman spectra of Bi_2Se_3 using no PTM (Run 2).

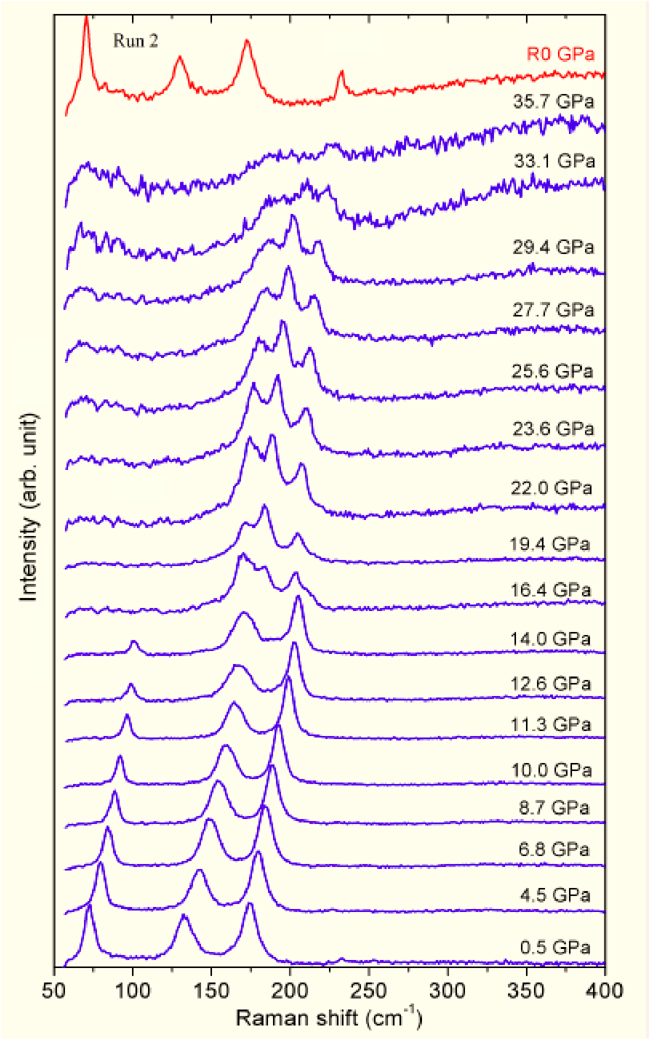

Fig. S6. Pressure dependence of Raman spectra of Bi_2Se_3 during compression and decompression (0 GPa) experimental using no PTM (Run 2).

(8) Pressure dependence of the Raman frequency and relative Raman shift of R-3m phase of Bi_2Se_3

The pressure dependence of the Raman frequencies for *R*-3*m* phases of Bi_2Se_3 is reported in Fig. 6. The Raman frequency of the three observed Raman peak from two independent Runs homogeneously increase as the pressure increases for the *R*-3*m* phase of Bi_2Se_3. However, the relative Raman shift of the *R*-3*m* phase of Bi_2Se_3 versus pressure is different, which is $A_{1g}^1 > E_g^2 > A_{1g}^2$. The difference between Run 1 and Run 2 lies in the profile of relative Raman shift, which come from the different hydrostatic conditions.

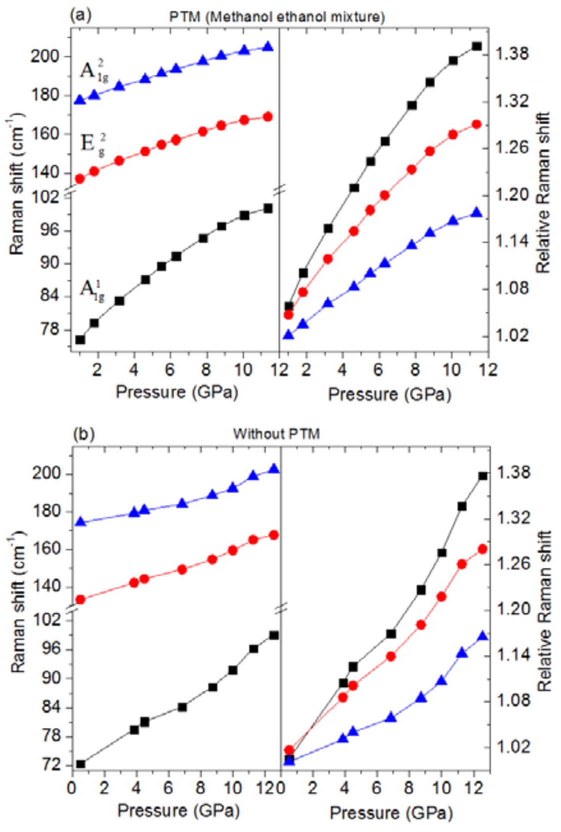

Fig. S7. Pressure dependence of the Raman frequency and relative Raman shift of R-3m phase of Bi_2Se_3 using (a) 4:1 methanol-ethanol mixture (Run 1) and (b) no PTM (Run 2).
